# Editorial: Evaluating differentiation therapy and biomarkers in myeloid malignancies

**DOI:** 10.3389/fonc.2026.1867602

**Published:** 2026-05-15

**Authors:** Nelida Ines Noguera, Syed Khizar Hasan, Laura Cicconi

**Affiliations:** 1 IRCCS Santa Lucia Foundation, Via del Fosso di Fiorano, Rome, Italy; 2 Department of Biomedicine and Prevention, Tor Vergata University, Rome, Italy; 3 Hasan Lab, Advanced Centre for Treatment, Research and Education in Cancer (ACTREC), Tata Memorial Centre, Navi Mumbai, India; 4 Department of Hematology, Polo Universitario Pontino, S.M. Goretti Hospital, Latina, Italy

**Keywords:** artificial intelligence, biomarkers, diagnostic, leukemia, myeloid malignancies, therapy

Driven by targeted treatment options, recent advances in molecular profiling, computational modelling and therapeutic innovation have reshaped the management of hematologic malignancies, enabling increasingly refined risk stratification and tailored treatments (1). Statistical approaches, based on algorithms governed by artificial intelligence (AI) methodologies, taking into account large sets of data on biomarkers, are redefining the diagnostic and therapeutic landscape of myeloid neoplasms (2, 3). *“Evaluating Differentiation Therapy and Biomarkers in Myeloid Malignancies”* brings together original research articles and reviews addressing key aspects of disease biology, diagnosis, and treatment.

Molecular, clinical, and computational data can be integrated to refine, sometimes redefine, disease classification, patient stratification, and guide therapeutic decision-making. Beyond myeloid malignancies, this is offering broader biological and translational insights relevant across hematologic diseases.

Relapse remains a major clinical challenge across hematologic malignancies, as illustrated by transcriptomic analyses in childhood B-cell precursor acute lymphoblastic leukemia (BCP-ALL). Despite survival rates exceeding 85%, relapse continues to account for a substantial proportion of treatment failure, including among patients initially classified as low risk. Transcriptomic signatures identified at diagnosis reveal intrinsic leukemic stem cells biology associated with relapse susceptibility, involving dysregulation of cell death and stress adaptation, immune and metabolic reprogramming, developmental plasticity and stemness, drug resistance, and post-transcriptional regulation (4). These findings underscore the limitations of current risk stratification approaches and support the integration of transcriptomic biomarkers for earlier and more accurate identification of high-risk disease. Notably, many of these biological programs are shared across hematologic malignancies, including myeloid neoplasms, where they contribute to disease persistence and therapeutic resistance.

In acute myeloid leukemia (AML), the role of maintenance therapy continues to be an area of active clinical investigation. A real-world cohort of 156 patients who achieved complete remission after induction therapy and consolidation with high dose cytarabine was evaluated to compare maintenance strategies using decitabine or conventional chemotherapy against a non-maintenance approach. Maintenance therapy was associated with significant improvements in both relapse-free survival and overall survival, and no overall differences were observed between decitabine and chemotherapy. Subgroup analyses indicated that clinical benefit was influenced by disease biology and treatment response, with greater efficacy in patients with intermediate-risk cytogenetics, absence of FLT3-ITD mutations, and early achievement of complete remission. Decitabine, in particular, demonstrated improved tolerability and favorable outcomes in patients receiving prolonged treatment, highlighting the importance of treatment duration and epigenetic modulation in maintaining disease control [Wei et al.]. These results support the concept that pharmacologic remodelling of leukemic cell states may represent a viable maintenance strategy in AML.

The increasing role of computational approaches is exemplified by advances in AI-assisted diagnostics in AML. Given the marked heterogeneity of the disease and the inherent limitations of conventional diagnostic workflows in terms of standardization, efficiency, and reproducibility, AI-based methods are emerging as powerful adjunct tools. Current applications span peripheral blood smear image analysis, flow cytometry interpretation, and genetic data modelling. AI has demonstrated the potential to improve diagnostic accuracy, reduce inter-observer variability, and uncover latent patterns relevant to disease characterization [Xie et al.]. In addition, these approaches may facilitate the discovery of novel biomarkers with diagnostic and prognostic relevance. However, challenges remain, particularly regarding generalizability across datasets, interpretability of models, and integration into clinical workflows. Overcoming these limitations will be essential for the successful translation of AI tools into precision hematology practice.

Biomarker discovery and systematization in myelodysplastic syndromes (MDS) further illustrates the power of integrating multi-omics data with machine learning and experimental validation. Using GEO datasets, differentially expressed genes were identified and analyzed through LASSO regression, SVM-RFE, and Random Forest algorithms, leading to the prioritization of key candidates including LDLRAD4, FAM43A, and KCNK5. Among these, LDLRAD4 emerged as a particularly relevant biomarker, showing strong associations with TGF-β and MAPK signaling pathways and a significant correlation with immune infiltration, especially natural killer (NK) cell populations. Functional studies further demonstrated that LDLRAD4 overexpression suppresses MDS cell proliferation, induces cell cycle arrest, and promotes apoptosis, suggesting a tumor-suppressive role in this context [Xu et al.]. These findings establish a direct link between molecular alterations and immune microenvironment dynamics, positioning LDLRAD4 as both a potential diagnostic/prognostic biomarker and a candidate therapeutic target. More broadly, this work highlights how the integration of bioinformatics, machine learning, and experimental validation can accelerate biomarker discovery in complex hematologic disorders.

Taken together, these studies illustrate the rapidly evolving landscape of hematologic malignancy research, where molecular profiling, epigenetic modulation, immune microenvironment characterization, and computational tools are becoming increasingly interconnected. A central theme emerging from this research trend is the shift from static classification systems toward dynamic, biology-driven frameworks that integrate disease state, immune context, and therapeutic response.

In conclusion, this Research Topic underscores the critical role of biomarkers, differentiation-based strategies, immune-informed molecular profiling, and AI-assisted diagnostics in advancing the field of hematologic malignancies (5). By bridging mechanistic insights with clinical and computational innovation, these studies collectively contribute to more precise, interpretable, and effective approaches for diagnosis and treatment.
